# Management of colorectal polyp cancers

**DOI:** 10.1308/003588412X13373405387771

**Published:** 2012-05

**Authors:** S Naqvi, S Burroughs, HS Chave, G Branagan

**Affiliations:** Salisbury NHS Foundation Trust,UK

**Keywords:** Colorectal neoplasm, Colonic polyp, Endoscopy

## Abstract

**INTRODUCTION:**

Management of malignant colorectal polyps is controversial. The options are resection or surveillance. Resection margin status is accepted as an independent predictor of adverse outcome. However, the rate of adverse outcome in polyps with a resection margin of <1mm has not been investigated.

**METHODS:**

A retrospective search of the pathology database was undertaken. All polyp cancers were included. A single histopathologist reviewed all of the included polyp cancers. Polyps were divided into three groups: clear resection margin, involved resection margin and unknown resection margin. Polyps were also analysed for tumour grade, morphology, Haggitt/Kikuchi level and lymphovascular invasion. Adverse outcome was defined as residual tumour at the polypectomy site and/or lymph node metastases in the surgical group and local or distant recurrence in the surveillance group.

**RESULTS:**

Sixty-five polyps (34 male patients, mean age: 73 years, range: 50–94 years) were included. Forty-six had clear polyp resection margins; none had any adverse outcomes. Sixteen patients had involved polyp resection margins and twelve of these underwent surgery: seven had residual tumour and two of these patients had lymph node metastases. Four underwent surveillance, of whom two developed local recurrence. Three patients had resection margins on which the histopathologist was unable to comment. All patients with a clear resection margin had no adverse outcome regardless of other predictive factors.

**CONCLUSIONS:**

Polyp cancers with clear resection margins, even those with <1mm clearance, can be treated safely with surveillance in our experience. Polyp cancers with unknown or involved resection margins should be treated surgically.

Polyp cancers in the colorectum are defined as adenomas within which an invasive carcinoma has developed and invaded by direct continuity through the muscularis mucosa into the submucosa.^1^ With the advent of the National Health Service (NHS) Bowel Cancer Screening Programme in 2006, the number of polyp cancers identified at colonoscopy in the UK has increased. The incidence of malignant colorectal polyps in the screening programme was 1.88% between 2006 and 2009.^2^ There is a debate over how to treat patients after endoscopic polypectomy for polyp cancer. The options are either a formal surgical resection or surveillance.^3,4^

Since the cancer has invaded into the submucosa, it has the potential to spread via lymphatics and blood vessels. The incidence of lymph node metastases in malignant polyps is around 6.7%.^3–15^ There is also the risk that having resected the polyp endoscopically, it may not have been adequately removed, leaving a risk of local recurrence.

It is widely accepted that resection margin status is a reliable prognostic factor in predicting adverse outcome in resected malignant polyps.^1,4–7,16^ However, most authors state that a clear resection margin is anywhere from 1mm^5^ to 2mm.^6^ We instituted a retrospective study of all patients with a proven polyp cancer over a ten-year period at Salisbury District Hospital to investigate whether a clear resection margin of any distance is associated with an adverse outcome.

## Methods

Cases of malignant colorectal polyps between March 2000 and September 2010 were identified retrospectively using the histology database. Our inclusion criteria were any macroscopic polypoid adenomas with a focus of carcinoma invading into the submucosa. Any cases in which dysplastic cells did not invade through the muscularis mucosae (high grade dysplasia) were excluded, as were polypoid cancers (ie a lesion with the macroscopic appearance of a polyp but constituting entirely malignant tissue when examined histologically). The notes for these cases were reviewed and data collected on polyp histology, outcomes, and the length and nature of follow-up.

Endoscopy reports for each patient were reviewed and the morphology of polyps was noted as either pedunculated or sessile. All specimens were re-examined by a single histopathologist at Salisbury District Hospital. Data were collected on resection margin, tumour grade, vascular invasion and level of invasion based on studies by Haggitt *et al*^14^ and Kikuchi *et al*.^15^

Patients were divided into four groups: i) resection margin clear by >1mm, ii) equivocal resection margin (0.1–1mm), iii) margin involved with tumour, and iv) unknown resection margin status (cases in which the histopathologist was unable to comment on the resection margin). Adverse outcomes were divided into those for patients who had surgery and those for patients who were treated conservatively with surveillance. Adverse outcomes in the surgical group were defined as presence of residual cancer at the site of polypectomy and/or lymph node metastasis. Those in the surveillance group were defined as local or distant recurrence. Follow-up data reviewed included length of follow-up, patient status and method of surveillance.

Statistical significance between the clear resection margin and involved resection margin groups was calculated using Fisher’s exact test.

## Results

Between March 2000 and September 2010, 68 patients were identified who had malignant colorectal polyps based on their original histology reports. After re-examination, three patients were excluded for not meeting the inclusion criteria (1 polypoid cancer and 2 high grade dysplasia). The remaining 65 polyps (33 male patients) with a mean age of 73 years (range: 50–93 years) fit the inclusion criteria.

Forty-six polyps (71%) were identified with cancer free resection margins. In this group, 21 (45%) had a resection margin of 0.1–1mm. Sixteen polyps (25%) had cancer involving the resection margin. Three polyps (5%) had resection margins on which the histopathologist was unable to comment.

Thirty patients (46%) underwent surgery with the remainder undergoing surveillance with a mean follow-up duration of 2.7 years (range: 0.5–5 years). Endoscopic surveillance was variable. However, all patients received at least a yearly colonoscopy until year two and then a three-yearly colonoscopy. Forty-one patients (63%) had staging computed tomography and only one revealed any spread (involved margin group).

In the group that received surgery, three patients (10%) had lymph node metastases and residual cancer was found in seven patients (23%). In the group that received surveillance, four patients (11%) had a local or distant recurrence.

**Figure 1:**
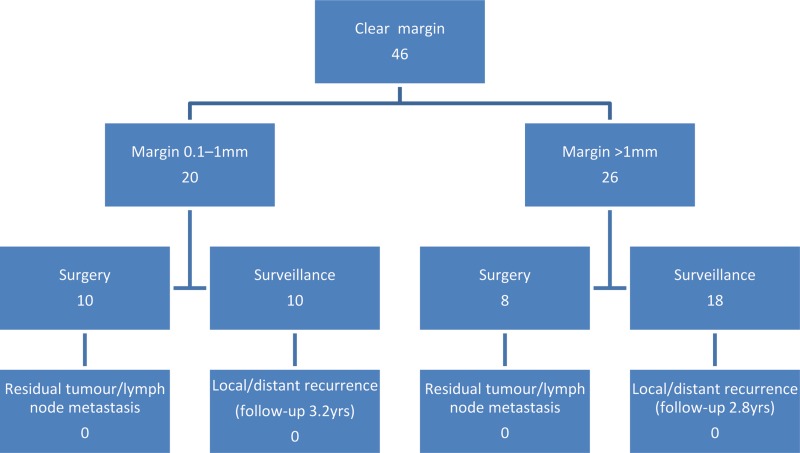
Flow diagram of polyp cancers with clear resection margins and rates of adverse outcome

Twenty-five patients (38%) had a resection margin of >1mm, seven of whom underwent surgery. None of these patients had residual tumour at the site of polypectomy on post-operative histological examination and none had involved lymph nodes. The remaining 18 patients underwent surveillance for a mean follow-up period of 2.8 years (range: 0.5–5 years) (Fig 1). Three have since died of causes unrelated to their malignancy. None of these patients had local or distant recurrence on follow-up. One patient was noted to have recurrent polyps at repeat colonoscopy but no evidence of malignancy.

Twenty-one patients (32%) had a resection margin of 0.1–1mm, eleven of whom underwent surgery. None of these patients had residual tumour at the site of polypectomy on post-operative histological examination and none had any involved lymph nodes. The remaining 10 patients underwent surveillance with a mean follow-up duration of 3.2 years (range: 0.5–5 years) (Fig 1). Two were lost to follow-up. The remainder have had no evidence of local or distant recurrence.

**Figure 2:**
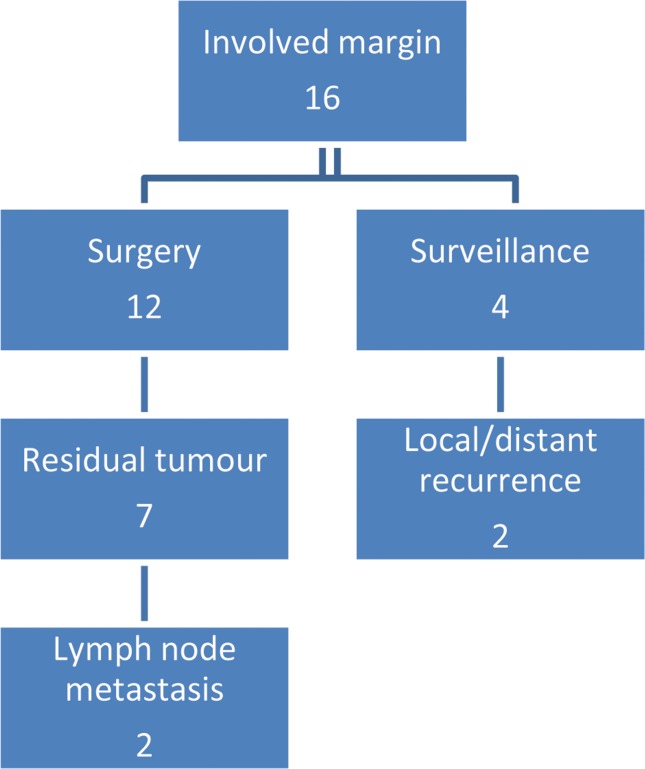
Flow diagram of polyp cancers with involved margins and rates of adverse outcome

Sixteen patients (25%) had resection margins involved at polypectomy (Fig 2). Twelve patients underwent surgery. Seven patients (44%) had residual tumour at the polypectomy site, three of whom had lymph node metastases. The remaining patients were free of residual cancer and lymph node metastases. Of the four patients who did not have surgery, two had adverse outcomes: one was deemed unfit for surgery and died of her disease, one declined treatment and died of her disease, one was unfit for surgery but had no evidence of recurrence at two years when he died of pulmonary fibrosis, and one is deceased but did not die of their disease.

The difference in adverse outcome between the clear margin groups overall and the involved margin group was statistically significant (Table 1).

**Table 1:** Comparison of clear versus involved resection margins

Resection margin	No adverse outcomes	Adverse outcomes	*P*-value
Clear	46	0	**<0.0001**
Involved	7	9	

Three patients (5%) had margins that the histopathologist was unable to assess. None of these patients underwent surgery. Two had significant co-morbidities precluding surgery. One patient who had a re-excision of her original polyp due to incomplete initial resection was found to have a local recurrence at one year and died of her disease. The second patient completed five years of follow-up with no recurrence. The third completed four years of follow-up with no recurrence.

Results were stratified against Haggitt and Kikuchi^14,15^ levels (Table 2). In the clear resection margin group, there was an even spread between all Haggitt and Kikuchi levels. However, there were no adverse outcomes regardless of Haggit/Kikuchi level. In the involved margin group, all recorded Haggitt/Kikuchi levels were either Haggitt 3 or 4 and associated with an even spread of adverse outcomes.

**Table 2:** Haggitt/Kikuchi levels versus adverse outcomes

	Haggit/Kikuchi 1	Haggit/Kikuchi 2	Haggit/Kikuchi 3	Haggit 4	Not noted
Clear margin	11	8	16	9	2
Adverse outcome	0	0	0	0	0
Involved margin	0	0	3	6	7
Adverse outcome	0	0	1 residual tumour	3 residual tumour	4 residual tumour
			1 recurrence		1 recurrence
Unknown margin	2	0	0	0	1
Adverse outcome	1 recurrence	0	0	0	0
Totals	13 (20%)	8 (12%)	19 (29%)	15 (23%)	10 (15%)

**Figure 3:**
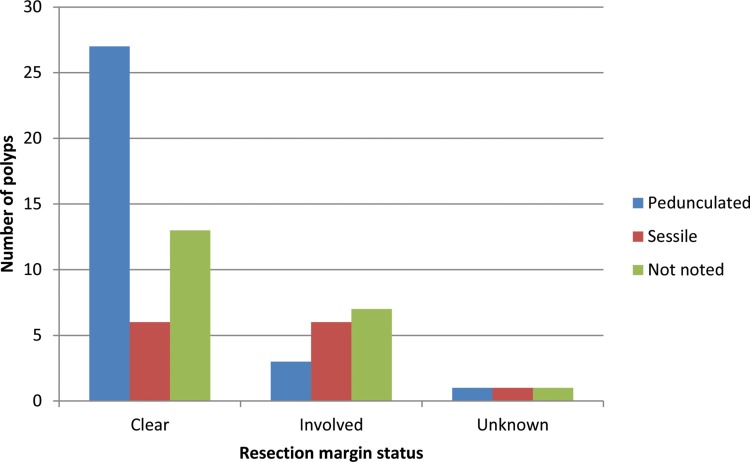
Graph comparing polyp morphology with resection margin status

Polyp morphology was divided into sessile, pedunculated or unknown as documented in endoscopy reports and subsequent histological examination (Table 3). Overall, there were 31 pedunculated polyps (47%), 13 sessile polyps (20%) and 21 (32%) whose morphology was not noted. Most polyps (52%) with a clear resection margin were pedunculated and most polyps in the involved margin group were either sessile or not noted (81%) (Fig 3). There were no adverse outcomes in the clear resection margin group regardless of polyp morphology.

**Table 3:** Polyp morphology versus adverse outcomes

	Clear	Adverse outcome	Involved	Adverse outcome	Unknown	Adverse outcome
Pedunculated	27	0	3	3	1	0
Sessile	6	0	6	4	1	0
Not noted	13	0	7	3	1	1

Vascular invasion (Table 4) was noted in ten cases (15%). Six of these patients were in the clear resection margin group and none had any adverse outcomes at either surgery or long-term follow-up. Three patients were in the involved margins group; two of these had residual tumour in their resected specimens at surgery.

**Table 4:** Vascular invasion versus adverse outcomes

	Vascular invasion	Adverse outcome
Clear margin	6	0
Involved margin	3	2 residual tumour
Unknown margin	1	0

We found only three patients (5%) with a poor grade tumour on histology (Table 5). One patient was in the clear margin group and had recurrent polyps but no malignancy. The remaining two were in the involved margin group and both had residual tumour in their resected specimens at surgery.

**Table 5:** Poor tumour grade versus adverse outcomes

	Poor grade	Adverse outcome
Clear margin	1	0
Involved margin	2	2
Unknown margin	0	–

## Discussion

Since the introduction of the NHS Bowel Cancer Screening Programme in 2006, an increasing number of polyp cancers have been identified as incidental findings at endoscopic polypectomy, which matches our own data. This highlights the need for a paradigm to decide which patients should be treated conservatively and which require surgical management.^3^

A number of histological and endoscopic parameters have been investigated to determine which resected polyps are at highest risk of lymph node metastasis and/or local recurrence. These include presence of carcinoma at the resection margin,^1,4–7,16^ morphology of the polyp,^14,15^ grade of carcinoma^4,7,11,13,14^ and presence of lymphovascular invasion.^4,6,17^

We investigated primarily the association between clear resection margin and adverse outcome. Resection margin status is widely accepted as an independent risk factor for adverse outcome.^1,4–7,16^ Our own data revealed 16 patients with involved margins, of whom 5 (31%) had an adverse outcome. None of these five had another positive predictor of adverse outcome. More strikingly, in the clear resection margin group, there were no adverse outcomes despite 15 specimens (23%) with 1 or more adverse risk factors (poor tumour grade, vascular invasion, Haggitt/Kikuchi 4).

Similarly, Netzer *et al* found that of 24 patients with involved margins, 9 (37.5%) had an adverse outcome and in 5 of these, resection margin status was the only risk factor.^6^ Seitz *et al* postulated that the use of diathermy to resect colorectal polyps induces post-diathermy necrosis in any residual tumour remnant.^16^ This would explain why there was no residual tumour in five patients with a positive resection margin.

A resection margin of 0.1–1mm is considered an involved margin and most studies in the literature would advise surgery. Cooper *et al* found that 21.4% of cases with cancer at or near the resection margin (0.1–1mm) had an adverse outcome but it was not clear how many of these had a measurable cancer–margin distance.^5^ Netzer *et al* noted, however, that all patients with a clearly cancer free resection margin, even those with a cancer–margin distance of <2mm, had no adverse outcome.^6^ Nevertheless, their study still advised a resection margin of ≥2mm as ‘safe’. Our own data found 20 patients with a resection margin of 0.1–1mm. None of these patients had an adverse outcome in either surgical or surveillance groups. This included nine cases (45%) with a resection margin of 0.1–0.5mm.

A study by the Northern Region Colorectal Cancer Audit Group on 386 polyp cancers supports this outcome.^17^ Their data suggested that a resection margin of >0mm in subsequently surgically resected specimens was significantly associated with no residual cancer. Tumour involvement at the excision margin was associated with residual tumour.

As with other studies looking at malignant colorectal polyps,^4^ the major limitation in our study was its retrospective nature. This is especially reflected in the group of patients in the unknown margin group. We acknowledge that no current multidisciplinary team setting would accept a report with no assessment of resection margin. These patients were included as resection margin status has been poorly noted historically.

All of the polyp cancers identified in our cohort were re-examined by a single histopathologist. The original histopathology reports for each specimen were also reviewed. These data revealed eight reports between 2000 and 2005 with no mention of resection margin status. In three of these reports the pathologist was unable to comment on the resection margin due to the alignment of the specimen. If the morphology of the polyps was compared with the resection margin status of the original reports, it was found that five (62%) of the eight patients with an unknown resection margin had sessile polyps whereas only six (13%) had sessile polyps in the clear resection margin group. The suggestion is that because sessile polyps are removed piecemeal at endoscopy, it is often difficult for the pathologist to identify the resection margin clearly. The fact that only three polyps had resection margins on which the histopathologist could not comment highlights that the situation is improving with experience.

In our study, polyp morphology itself did not affect the rate of adverse outcome. Haggit *et al* postulated that all sessile polyps were high risk and were assigned level 4 in their classification.^14^ However, Kikuchi *et al* noted that 32 of 105 sessile polyps were level Sm1 in their classification system and had the same risk of adverse outcome as all other Sm1 polyps.^15^ Our data support this and other studies^4^ in that there was a higher proportion of sessile polyps in the high risk involved margin group but if the resection margin was clear, they should be treated as any other polyp with a clear resection margin.

Ten specimens with vascular invasion were found in our dataset. Six (60%) of these were in the clear resection margin group and had no adverse outcomes. Three (30%) were in the involved margin group and two (66%) of these had an adverse outcome. This suggests vascular invasion is a significant risk factor for adverse outcome in polyp cancers although our numbers were too small to draw any firm conclusions. Evidence in the literature suggests that vascular invasion does not correlate well with outcomes in polyp cancers and is therefore of poor prognostic value.^4,18^

Poorly differentiated tumours are rare in polyp cancers. We found only three specimens (4%). In our literature search, the mean incidence was 3.1%.^4,7,11,13,14^ Some studies have postulated that it is a significant risk factor on account of its aggressive nature ^4,5,7^ but it has not been found to be significant in around half the studies.^6,15^ In our dataset, poorly differentiated tumour grade was not associated with adverse outcome in the clear resection margin group. However, two poorly differentiated tumours were found in the involved margin group and both resulted in adverse outcomes. Further research is needed on a large cohort of polyps with poorly differentiated tumours before firm conclusions can be reached.

## Conclusions

In our study resection margin status is an independent risk factor for adverse outcome. A polyp cancer with an involved resection margin should be considered for surgical resection. A clear resection margin (of any distance, even those ≤1mm) can be considered low risk and therefore managed non-surgically.

The incidence of polyp cancers is set to rise with increased numbers being identified in the bowel cancer screening programme and, consequently, a clear treatment algorithm needs to be devised to treat patients correctly and safely. Polyp specimens removed piecemeal are much more difficult to interpret by the histopathologist and have the potential to lead to a resection margin status that is difficult or impossible to assess. The aim of polypectomy should therefore be to remove the specimen intact.
